# A Case of Gun Shot Wound of the Back, Producing Paralysis, Relieved by Laminectomy

**Published:** 1915-05

**Authors:** T. C. Davison

**Affiliations:** Adjunct Professor of Surgery, Atlanta Medical College; Associate Surgeon to Grady Hospital and Visiting Surgeon to Georgia Baptist Hospital, Atlanta, Ga.


### A CASE OF GUN SHOT WOUND OF THE BACK, PRODUCING PARALYSIS, RELIEVED BY LAMINECTOMY.*

By T. C. Davison, M. D., Adjunct Professor of Surgery, Atlanta Medical College; Associate Surgeon to Grady Hospital and Visiting Surgeon to Georgia Baptist Hospital, Atlanta, Ga.

The following case offers unusual features of clinical interest:

A negro, 18 years of age, was shot by a policeman, October 10th, 1914, and was brought to the hospital with complete paralysis of the bladder, bowels and both legs. The patient came under my observation four weeks after receiving the wound. His chart history was meagre, incomplete and misleading, as follows:

"Patient admitted to hospital with gunshot wound of right Scapular region, X-Ray shows bullet on right side opposite 2nd lumbar vertebra."

According to this the paralysis could not be accounted for. The X-Ray had been misread, the bullet was on the left side, having crossed over and in so doing had passed through the body of 2nd lumbar vertebra at an angle of 45 degrees downward and to the left. It was shown imbedded in the muscle about one and a half inches from spinous processes of lumbar vertebrae, opposite intervertebral cartilage between 2nd and 3rd lumbar vertebrae. The question arose, was the cord injured by the bullet, or is it compressed by fractured bone, bullet or blood clot?

The following interesting history was secured which aided me in making a diagnosis, and upon the strength of it I operated and relieved the paralysis.

History: He was shot by a policeman who was standing upon the steps of a house about four feet above the ground, ten feet to the right and a little to the rear of the patient. After being shot, the patient remained standing during a period of two or three minutes, when his legs gave away and he fell, having lost control of muscles of legs, though sensation remained good.

The patrol was called and he was taken to the hospital. His legs became numb and heavy, when he reached there he had no feeling in his legs at all. This was probably 30 minutes after he was shot.

This history is very interesting, and clearly shows that the bullet did not sever the cord as the patient still was able to stand for two or three minutes. Also for same reason the compression which came on gradually could not have been caused by the bullet or piece of fractured bone, but by a gradually forming blood clot in the spinal canal, which first pressed upon and put out of commission the anterior motor roots and later by continued enlargement and pressure the posterior sensory roots, showing that the blood clot was in front of the cord.

I operated November 11, 1914. An incision was made in the median line over the spinous processes of lumbar vertebrae. The muscles were separated and retracted, the spinous processes and lamina of 2nd and 3rd lumbar vertebra were removed. The coverings of the cord were examined and appeared normal; the dura was incised longitudinally, allowing the cerebro spinal fluid to escape; it gushed out as if under considerable pressure. The tip of the cord and nerves were examined, and there was no blood clot inside the dura. The pressure was believed to have been caused by a clot in front of the dura behind bodies of vertebrae. The dura was sutured, wound closed without drainage, and healed by first intention. The patient showed considerable shock, but reacted nicely. The next morning he showed considerable improvement and he rapidly regained control of legs, bladder and bowels. The patient was up in fourteen days and now I present him to you a well man.

The operation had the effect of a decompression operation by removing the posterior part of bony circle against which the cord was compressed. This case shows the importance of an accurate history and proper reading of X-Ray plates as well as a thorough examination of the patient.

908 Empire Life Building, Atlanta, Ga.

